# Supplementary material for: The impact of tinnitus on adult cochlear implant recipients: A mixed-method approach
Source: PLoS One. 2023 Apr 20;18(4):e0284719. doi: 10.1371/journal.pone.0284719 (PMC10118117; doi:10.1371/journal.pone.0284719)
Supplement: S1 Table — (PDF) [file pone.0284719.s001.pdf]

	Question	Response type
0	Are you experiencing tinnitus?	Multiple choice (x4)
1a	Do you use a hearing aid?	Binary (yes/no)
1b	[IF YES] On which side do you wear your hearing aid?	Multiple choice (x2)
2	On which side do you wear your sound processor(s)?	Multiple choice (x3)
3	Which type of hearing loss do you have?	Multiple choice (x3)
4	What best describes your tinnitus?	Multiple choice (x2)
5	Where do you hear your tinnitus?	Multiple choice (x7)
6a	Did you have tinnitus before implantation?	Multiple choice (x3)
6b	[IF YES] How much of a problem was your tinnitus before implantation?	Multiple choice (x5)
7a	Did your tinnitus change after implantation?	Multiple choice (x3)
7b	[IF YES] How did it change?	Multiple choice (x7)
8	How many hours per day do you wear your sound processor?	Numerical text
	Please explain if it varies day to day.	Open field text
9	What percentage of your time awake are you consciously AWARE OF your tinnitus?	Numerical rating scale
10	What percentage of your time awake are you ANNOYED by your tinnitus?	Numerical rating scale
11	When you are wearing your sound processor, how much of a problem is your tinnitus?	Multiple choice (x5)
12	When you are not wearing your sound processor, how much of a problem is your tinnitus?	Multiple choice (x5)
13	In general, how much of a problem is your tinnitus?	Multiple choice (x5)
14	Does your tinnitus make it difficult to fall asleep or stay asleep?	Numerical rating scale (x2)
15	Does your tinnitus make you feel tired?	Numerical rating scale (x2)
16	Does your tinnitus make you feel stressed?	Numerical rating scale (x2)
17	Does your tinnitus make you feel depressed?	Numerical rating scale (x2)
18	Does your tinnitus make you feel anxious?	Numerical rating scale (x2)
19	Does your tinnitus make you angry?	Numerical rating scale (x2)
20	Does your tinnitus make it difficult to concentrate?	Numerical rating scale (x2)

21	Does your tinnitus make it difficult to work or perform other tasks such as home maintenance, schoolwork, or caring for children or others?	Numerical rating scale (x2)
22	Does your tinnitus make it difficult to hear clearly?	Numerical rating scale (x2)
23	Does your tinnitus make it difficult to listen to radio or television?	Numerical rating scale (x2)
24	Does your tinnitus make it difficult to follow conversations in a group or in meetings?	Numerical rating scale (x2)
25	Does your tinnitus interfere with your ability to enjoy social activities?	Numerical rating scale (x2)
26	Does your tinnitus cause or aggravate other health problems?	Multiple choice (x3)
27	How do the following situations affect your tinnitus?	Numerical rating scale (x10)
28a	Are there any other situations where your tinnitus gets better?	Binary (yes/no)
28b	[IF YES] Please provide details	Open field text
29a	Are there any other situations where your tinnitus gets worse?	Binary (yes/no)
29b	[IF YES] Please provide details	Open field text
30a	What strategies/techniques do you use to manage your tinnitus during the day?	Multiple choice (x9)
30b	What effect does this strategy/technique have on your tinnitus?	Numerical rating scale
31a	What strategies/techniques do you use to manage your tinnitus when you want to sleep?	Multiple choice (x6)
31b	What effect does this strategy/technique have on your tinnitus?	Numerical rating scale
32a	Which treatments have you had or are currently receiving to manage your tinnitus?	Multiple choice (x7)
32b	What effect does this treatment have on your tinnitus?	Numerical rating scale
33	How easy is it to manage your tinnitus in general	Numerical rating scale (x2)
