# Supplementary material for: The impact of tinnitus on adult cochlear implant recipients: A mixed-method approach
Source: PLoS One. 2023 Apr 20;18(4):e0284719. doi: 10.1371/journal.pone.0284719 (PMC10118117; doi:10.1371/journal.pone.0284719)
Supplement: S2 Table — (PDF) [file pone.0284719.s002.pdf]

Theme	Sub-themes	Codes
Tinnitus experience	Awareness/Annoyance	Always bothered
		Bothered intermittently
		Always aware, not bothered
	Dependency of the sound processor use	Aways aware, only bothered when not wearing the sound processor
		Only aware when not wearing the sound processor
	Dependency on the implantation side	Aware in the non-implanted ear
Situations impacting tinnitus	Bedtime	When going to sleep or sleeping
		When waking up
	Environmental change	During extreme change in weather
		During change in atmospheric pressure
	Mental state	When being anxious
		When being stressed
		When being mentally tired
		After a concentration effort
		When bringing attention to tinnitus
	Physical state	When being physically tired
		After intense physical effort
		When being sick
	Sound environment	When being in a quiet environment
		When being in a loud or noisy environment
		During a hearing test or a CI programming session
		During group conversations
		During auditory overstimulation
	Sound processor status	When the sound processor is on
		When the sound processor is off
Tinnitus-related difficulties	Auditory-related difficulties	Communication difficulties
		Hearing difficulties
		Sensitivity to sounds
	Comorbidities worsening	Dizziness
		Hyperacusis
		Migraine
		Pain
	Concentration difficulties	

	Difficulties at work	
	Fatigue	
	Sleep disorders	
	Psychological problems	Anxiety
		Angriness
		Depression
		Stress
	Social isolation	
Tinnitus management strategies	Change in CI use	Turn the sound processor on
		Turn the sound processor off
		Wear the sound processor while sleeping
	Change in CI settings	Change volume
		Change sensitivity
		Personalized CI fitting
	Self-performed avoidance strategies	Isolation
		Avoid noise environment
		Avoid physical effort
	Self-performed distraction activities	Reading
		Listening to TV or music
		Walk
		Physical activity
	Self-performed stress management	Breathing exercises
		Mindfulness, meditation
		Relaxation
		Sophrology
		Sleep or rest
	Therapies provided by professionals	Behavioral therapy
		Group support
		Cranial massages
		Hearing or sound therapy
		Homeopathy
		Osteopathy
		Failure of care
