# Supplementary material for: The impact of tinnitus on adult cochlear implant recipients: A mixed-method approach
Source: PLoS One. 2023 Apr 20;18(4):e0284719. doi: 10.1371/journal.pone.0284719 (PMC10118117; doi:10.1371/journal.pone.0284719)
Supplement: S3 Table — All comorbidities are extracted from an open field question and grouped into similar themes. (PDF) [file pone.0284719.s003.pdf]

**Comorbidities**	**N (%)**<br>(*n* = 80)
Anger	1 (1.25)
Anxiety	6 (7.5)
Balance disorders	12 (15.0)
Cold symptoms	3 (3.7)
Concentration difficulties	3 (3.7)
Depression	10 (12.5)
DFNA9 symptoms	1 (1.25)
Fatigue	10 (12.5)
Hearing difficulties	2 (2.5)
Hyperacusis	1 (1.25)
Hypertension	6 (7.5)
Migraine	11 (13.8)
Meniere’s attack	2 (2.5)
Muscle tension	1 (1.25)
Neck pain	6 (7.5)
Psychosomatic complaint	1 (1.25)
Personality disorder	1 (1.25)
Sadness	1 (1.25)
Shoulder pain	2 (2.5)
Sleep disorders	8 (10.0)
Social isolation	1 (1.25)
Stress	11 (13.8)
Teeth grinding	1 (1.25)
Temporomandibular joint disorder	1 (1.25)
