# Supplementary material for: The impact of tinnitus on adult cochlear implant recipients: A mixed-method approach
Source: PLoS One. 2023 Apr 20;18(4):e0284719. doi: 10.1371/journal.pone.0284719 (PMC10118117; doi:10.1371/journal.pone.0284719)
Supplement: S1 Dataset — (ZIP) [file pone.0284719.s004.zip › Minimal data set/Cognitive interview probe dd 06.07.2021.docx]

**Interview probe** (Cognitive interview)

Thanks for your participation. The aim of the interview is to test the content of the survey.

There will be two parts:

- during the first part, I will ask you to read each question of the survey out loud and reply to it orally. It means that I would like you to tell me everything you think about as you work through each question. You will do this one question at the time.
- during the second part, after you filled the entire survey, I will ask you some complementary questions.

If there are questions you don’t understand, you can also ask me directly.

1. Do you agree to record the interview?

Answer:

1. Could you describe what this questionnaire is about?

Answer:

1. Did you understand the instructions for filling out the questionnaire?

Answer:

1. Were the questions clear for you?

If not, which questions were unclear and why?

Answer:

1. Do you feel there were any topics or aspects of tinnitus difficulties that were missed by the questionnaire?

Answer:

1. What did you think of the way the questions are posed and the answer options?

Answer:

1. (*optional in case of a hesitation in filling out a specific question)*Could you explain what question (…) is about?

Answer:

1. Do you have any suggestions to improve the questionnaire?

Answer:

Specific questions:

Question 3 & 4: how do you make the difference between question 3 and 4?

Answer:

Question 7: how do you understand the word ‘in general’?

Answer:

Question 8 & 9: how do you make the difference with the question 7?

Answer:

Question 21: what was going through your mind as you tried to answer the question 21?

Answer:

Question 25: Was there something unclear in this question?

Answer:

Will you understand if you ask instead: what the most important strategies you use? Rank them in order of importance?

Answer:

Question 27: Was there something unclear in this question?

Answer:

Question 28: what was going through your mind as you tried to answer the question 28?

Answer:
